# Phylogenetically Novel Uncultured Microbial Cells Dominate Earth Microbiomes

**DOI:** 10.1128/mSystems.00055-18

**Published:** 2018-09-25

**Authors:** Karen G. Lloyd, Andrew D. Steen, Joshua Ladau, Junqi Yin, Lonnie Crosby

**Affiliations:** a Department of Microbiology, University of Tennessee, Knoxville, Tennessee, USA; b Department of Earth and Planetary Sciences, University of Tennessee, Knoxville, Tennessee, USA; c Gladstone Institutes, University of California, San Francisco, San Francisco, California, USA; d Joint Institute for Computational Sciences, University of Tennessee, Knoxville, Tennessee, USA; University of Waterloo

**Keywords:** environmental microbiology, phylogeny, uncultured microbes

## Abstract

To describe a microbe’s physiology, including its metabolism, environmental roles, and growth characteristics, it must be grown in a laboratory culture. Unfortunately, many phylogenetically novel groups have never been cultured, so their physiologies have only been inferred from genomics and environmental characteristics. Although the diversity, or number of different taxonomic groups, of uncultured clades has been studied well, their global abundances, or numbers of cells in any given environment, have not been assessed. We quantified the degree of similarity of 16S rRNA gene sequences from diverse environments in publicly available metagenome and metatranscriptome databases, which we show have far less of the culture bias present in primer-amplified 16S rRNA gene surveys, to those of their nearest cultured relatives. Whether normalized to scaffold read depths or not, the highest abundances of metagenomic 16S rRNA gene sequences belong to phylogenetically novel uncultured groups in seawater, freshwater, terrestrial subsurface, soil, hypersaline environments, marine sediment, hot springs, hydrothermal vents, nonhuman hosts, snow, and bioreactors (22% to 87% uncultured genera to classes and 0% to 64% uncultured phyla). The exceptions were human and human-associated environments, which were dominated by cultured genera (45% to 97%). We estimate that uncultured genera and phyla could comprise 7.3 × 10^29^ (81%) and 2.2 × 10^29^ (25%) of microbial cells, respectively. Uncultured phyla were overrepresented in metatranscriptomes relative to metagenomes (46% to 84% of sequences in a given environment), suggesting that they are viable. Therefore, uncultured microbes, often from deeply phylogenetically divergent groups, dominate nonhuman environments on Earth, and their undiscovered physiologies may matter for Earth systems.

**IMPORTANCE** In the past few decades, it has become apparent that most of the microbial diversity on Earth has never been characterized in laboratory cultures. We show that these unknown microbes, sometimes called “microbial dark matter,” are numerically dominant in all major environments on Earth, with the exception of the human body, where most of the microbes have been cultured. We also estimate that about one-quarter of the population of microbial cells on Earth belong to phyla with no cultured relatives, suggesting that these never-before-studied organisms may be important for ecosystem functions.

**Author Video**: An author video summary of this article is available.

## Abstract (precis)

In the past few decades, it has become apparent that most of the microbial diversity on Earth has never been characterized in laboratory cultures. We show that these unknown microbes, sometimes called “microbial dark matter,” are numerically dominant in all major environments on Earth, with the exception of the human body, where most of the microbes have been cultured. We also estimate that about one-quarter of the population of microbial cells on Earth belong to phyla with no cultured relatives, suggesting that these never-before-studied organisms may be important for ecosystem functions.

## INTRODUCTION

Direct sequencing of environmental DNA has shown that most microbial lineages have not been isolated in pure culture (1–3). However, the cellular abundances and viability states of uncultured microbes at different levels of phylogenetic divergence from their closest cultured relative are unknown. Because greater phylogenetic distance correlates with higher levels of evolutionary changes, uncultured groups may have novel undiscovered functions. Cellular abundance and viability may, in some cases, signify importance with respect to current ecosystem functions, in contrast to the members of the “rare biosphere” that become important for ecosystem functioning when conditions change (4). With the exception of keystone species, which can have great ecosystem importance even at low biomass concentrations, prokaryotic abundance and viability are generally indicators for participation in current ecosystem functions (1).

Quantifying the cellular abundance of all microbial taxa in any sample is challenging. Fluorescent *in situ* hybridization (FISH) allows fluorescent tagging of a taxonomic group, whose cells can then be counted under a microscope (2). However, FISH requires developing probes for phylogenetic groups one by one, which is impractical for quantifying highly diverse natural samples that are often comprised of thousands of species (3). Furthermore, FISH techniques are not always quantitative in all environments, due to taxon-specific biases in probe efficacy (4, 5). Quantitative PCR has the same low-throughput limitations, because individual measurements must be made for each taxon, and primer bias makes them not absolutely quantitative (4). However, understanding the total cellular abundance of uncultured clades of archaea and bacteria in all environments on Earth is important to the field of microbiology, so we approximated it using the data available in public databases.

Genes encoding the 16S rRNA small subunit of the ribosome are the most commonly used taxonomic and phylogenetic identifiers for bacteria and archaea, and most scientific journals make publication contingent on the deposition of 16S rRNA gene sequences into public databases. Therefore, the National Center for Biotechnology Information (www.ncbi.nlm.nih.gov) houses a nearly complete database of full-length 16S rRNA gene sequences. This database is subject to biases because the gene entries have undergone exponential amplification from their initial abundances, and small mismatches between DNA primers and different taxa are magnified during this amplification (5). Nevertheless, we examined this database here because it incorporates microbial phylogenetic information from thousands of different research studies. Assembled metagenomes provide a less biased accounting of 16S rRNA genes from a given environment. For such analyses, all DNA is chemically extracted from a sample, purified, sequenced in a small-read high-throughput platform, and then bioinformatically assembled into contigs. Full-length 16S rRNA genes can be identified in these contigs using hidden Markov model-based programs such as RNAmmer (6). If the sequencing depth is great enough, quantifying read recruitment to each 16S rRNA gene provides the best relative quantification of individual 16S rRNA genes currently available.

Cellular activity, however, is as important to environmental functions as cellular abundance (1). In cultured cells, rRNA content correlates with cellular activity (7), although no universally predictive relationship between those two parameters has been identified (8). Metatranscriptomes, in which 16S rRNA transcripts are converted to cDNA and sequenced without the use of primers, provide an estimate of which cells contained ribosomes and were therefore at least poised for activity in the environment (8).

We determined the identity of nearly all 16S rRNA gene sequences from public databases, to get a first estimate of the global abundance of microbial clades at different levels of similarity to their nearest cultured relative in different environments. The metagenomic and metatranscriptomic data sets show that uncultured clades dominate the cellular abundance of nonhuman Earth environments. Knowing the global abundance of cells from uncultured taxa is crucial for estimating the importance of uncultured lineages to ecosystem functions, determining the appropriateness of using cultured microbes as model systems for natural environments, and predicting the causes of unculturability.

## RESULTS AND DISCUSSION

**FIG 1:**
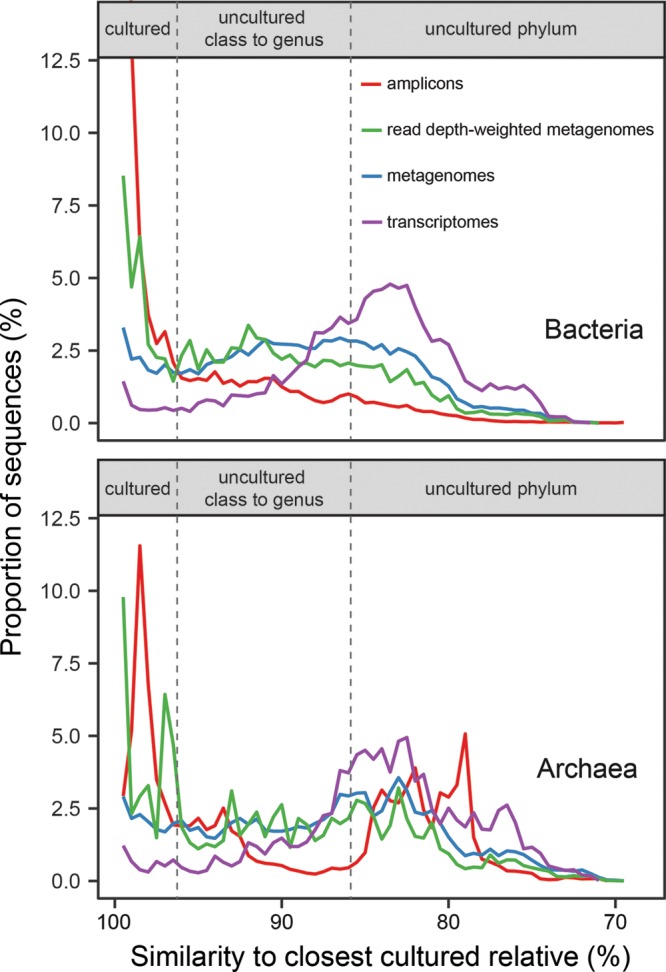
Fractions of 16S rRNA genes from bacteria (top panel) and archaea (bottom panel) in public databases from primer-amplified metagenomes (with and without read depths) and metatranscriptomes at different percent identities with their closest cultured relative. Vertical dashed lines represent estimated cutoff levels for different taxonomic levels of novelty relative to all cultures (indicated at the top of the panel) (9). Primer-amplified bacterial sequences showed 30% to up to 100% similarity to their closest cultured relative but were removed for clarity.

More than a third of primer-amplified 16S rRNA gene sequences were from the same species or genus as a culture (37% for bacteria and 34% for archaea; Fig. 1), in agreement with previous findings indicating that primer-amplified databases skew toward cultured organisms (9–11). However, even in the primer-amplified data set, the majority of sequences were from uncultured genera or higher taxonomic groups, including 17% and 44% from uncultured phyla in bacteria and archaea, respectively. This suggests that, considering all full-length 16S rRNA genes in public databases as a group, uncultured microbes, including those that are very highly divergent, are fairly abundant. Metagenomes had lower fractions of 16S rRNA gene sequences from cultured species (Fig. 1), with 15% for both bacteria and archaea based on total sequences and 28% for bacteria and 31% for archaea based on scaffold read depths. The rest of the 16S rRNA gene sequences were from uncultured genera and higher taxonomic groups, with about one-third of total sequences from uncultured phyla (36% and 46% without read depths and 24% and 33% with read depths for bacteria and archaea).

We recognize that it is impossible to absolutely link 16S rRNA gene identity to taxonomic level, because phylogenetic difference is inconsistently related to 16S rRNA gene sequence difference across lineages (12). These sequence similarity cutoff levels are proxies for degrees of phylogenic novelty rather than rigidly defined taxonomic levels. By using published values for similarity bins (12), our findings are comparable to those of other studies. Therefore, 16S rRNA gene sequences from uncultured cells were more abundant than those from cultured cells, suggesting that uncultured microbial clades are not relegated solely to the rare biosphere (13) but are instead numerically dominant.

We found that highly divergent uncultured sequences were better represented in metatranscriptomes than in metagenomes, with only 4% (bacteria) and 5% (archaea) of total sequences from cultured species to genera and 65% (bacteria) and 71% (archaea) of total sequences from uncultured phyla. Therefore, cells from highly divergent uncultured groups were alive *in situ*. However, the greater abundance of uncultured clades in metatranscriptomes than in metagenomes signifies a greater per-cell number of ribosomes, because all of the data at the Joint Genome Institute (JGI) undergo rRNA depletion, which uses primers to retrieve well-known ribosomal sequences. The sequences are proprietary, but they are almost certainly based on cultured organisms, which would bias the remaining sequences to include a higher proportion of uncultured clades. However, a comparison between metagenomes and metatranscriptomes, both of which were derived from the same samples in the Gulf of Mexico, showed that uncultured clades were indeed active relative to cultured clades (14).

**FIG 2:**
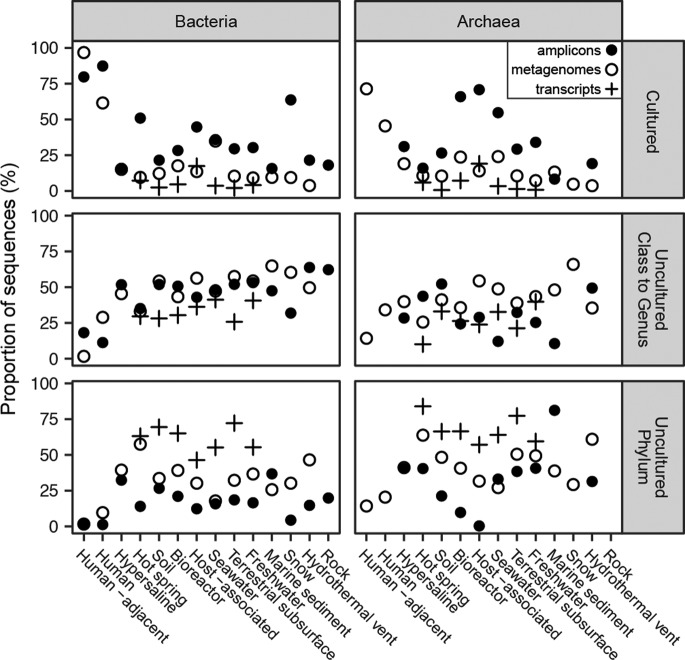
Proportion of 16S rRNA gene sequences in each category of phylogenetic novelty relative to cultures for each environment, by amplicons, metagenomes (without scaffold read depth), and metatranscriptomes. Closed circles represent primer-amplified amplicons, open circles represent metagenomes, and crosses represent transcriptomes. Total numbers of sequences and studies are listed in Table S2.

Contributions from uncultured clades varied by environment (Fig. 2). The only environments dominated by sequences from cultured species and genera were the human body and human-adjacent environments (Fig. 2). This result was not due to primer bias, because primer-amplified and metagenomic data sets contained mostly cultured species and genera (45% to 97%, inclusive of bacteria and archaea). High culturability in human environments likely benefits from a high frequency of culturing efforts, because all culturing happens in the vicinity of humans, and the study of human diseases has driven much research (15). Uncultured clades were also present in humans and human-adjacent environments, but very few were uncultured at taxonomic cutoff levels above the family level.

**FIG 3:**
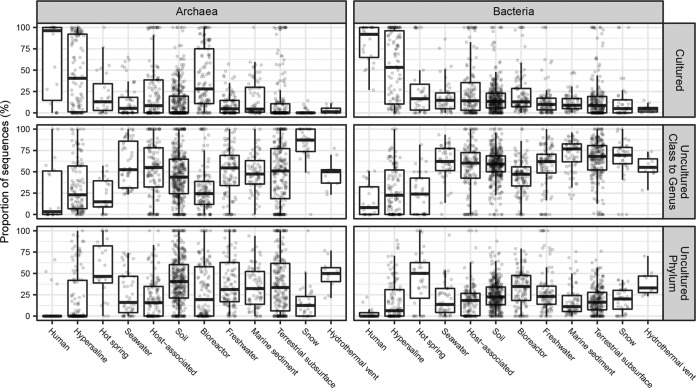
Proportion of 16S rRNA gene sequences by scaffold read depth averaged across all metagenomes. Each single data point represents the abundance of reads in that similarity bin from a single metagenome. Rows represent different similarity bins.

Primer bias toward cultures was more severe in all other environments, where uncultured archaea and bacteria were much more abundant in metagenomic data sets than in primer-amplified data sets (Fig. 2). Archaea in marine sediments represented an exception, possibly indicating that commonly used primers have good matches to the uncultured phyla that are abundant in these environments (16). To avoid primer bias and account for a high environmental abundance of closely related sequences, we used the metagenomic data sets with read depths to estimate quantifications (Fig. 3). Hypersaline environments were the next-best-cultured environments after human environments, with nearly half of archaea and bacteria being from cultured genera and very few from uncultured phyla (Fig. 3). The next-best-cultured group consisted of archaea in bioreactors. All other environments had more sequences from uncultured phyla than from cultured genera. Hot springs and hydrothermal vents, in particular, had high frequencies of uncultured phyla identified as both bacteria and archaea. Even though human host environments were dominated by cultured groups, nonhuman hosts had as few sequences from cultured archaea and bacteria as soil, seawater, freshwater, marine sediment, terrestrial subsurface, snow, and bioreactors did (for bacteria). This suggests that highly divergent uncultured microbes, possibly with novel functions, dominate nonhuman environments on Earth.

By using a large collection of publicly available sequences that represent as complete a sampling as possible, our sequence abundance quantifications can be extrapolated to global cell estimates, although this approach is biased against cells that are less amenable to DNA extraction and undersampled environments. Copy numbers of 16S rRNA genes per cell can be determined only for completed genomes (means of 3.8 copies/genome for 1,657 bacterial genomes and 1.8 copies/genome for 79 archaeal genomes on the IMG database (https://img.jgi.doe.gov/mer/; accessed 30 March 2018). However, only a few closed genomes are currently available for uncultured organisms (17). Applying the 16S rRNA gene copy numbers for completed genomes to our estimations of total cells would increase our estimates of the abundance of uncultured organisms, because estimations of archaea, which we found to be less well cultured, would be divided by the smaller number. Therefore, we use the conservative simplification of a single 16S rRNA gene copy number per genome to estimate that 81% (7.3 × 10^29^ cells) of microbial cells on Earth are from uncultured genera or higher and 25% (2.2 × 10^29^ cells) are from uncultured phyla (Table 1). Deriving abundance data from metatranscriptomes, the number of uncultured cells increased to 98% (5.9 × 10^29^), with uncultured phyla contributing 69% (4.2 × 10^29^) (Table 2). If the terrestrial subsurface data sets lack contributions from the ultrasmall uncultured cells missed in standard filtering methods (18), or if DNA extraction favors cultured taxa, which may have more easily lysed cell membranes, then these values represent underestimates of the abundance of uncultured cells on Earth.

**TABLE 1:** Metagenome-based estimates of global microbial cell abundances from uncultured archaea and bacteria, based on 16S rRNA gene sequence read depths^*a*^

Environment (reference)	No. (%) of microbial cells × 10^26^^*b*^			
	Total	Cultured species to genera	Uncultured genera to classes	Uncultured phyla and higher
Marine sediment (48)	2,900	390 (13)	1,921 (66)	590 (20)
Soil (49)	2,560	454 (18)	1,268 (50)	839 (33)
Terrestrial subsurface (49)	2,500	702 (28)	1,211 (48)	587 (23)
Seawater (49)	1,010	143 (14)	640 (63)	229 (23)
Freshwater (49)	1.3	0.1 (11)	0.8 (64)	0.3 (25)
Plant hosts (50)	1	0.5 (49)	0.4 (37)	0.1 (14)
Animal hosts (51)	0.2	0.1 (49)	0.1 (37)	0.0 (14)
				
Total	8,974	1,689 (19)	5,050 (56)	2,245 (25)

a Environments with fewer microbial cells were excluded.

b Cutoff values represent the upper 95% confidence interval of the median 16S rRNA gene identity for each taxonomic level (12).

**TABLE 2:** Metatranscriptome-based estimates of global microbial cell abundances from uncultured archaea and bacteria, based on 16S rRNA gene sequence numbers^*a*^

Environment (reference)	No. (%) of microbial cells × 10^26^^*b*^			
	Total	Cultured species to genera	Uncultured genera to classes	Uncultured phyla and higher
Marine sediment (48)	NA	NA	NA	NA
Soil (49)	2,560	49 (2)	758 (30)	1,753 (69)
Terrestrial subsurface (49)	2,500	45 (2)	597 (24)	1,858 (74)
Seawater (49)	1,010	36 (4)	389 (38)	587 (58)
Freshwater (49)	1.3	0.0 (3)	0.5 (40)	0.7 (56)
Plant hosts (50)	1	0.2 (18)	0.3 (33)	0.5 (49)
Animal hosts (51)	0.2	0.0 (18)	0.1 (33)	0.1 (49)
				
Total	6,074	129 (2)	1,744 (29)	4,200 (69)

a Environments with fewer microbial cells were excluded.

b Cutoff values represent the upper 95% confidence interval of the median 16S rRNA gene identity for each taxonomic level (12). NA, not applicable (too few metatranscriptome data are available from the indicated environment to be included).

**FIG 4:**
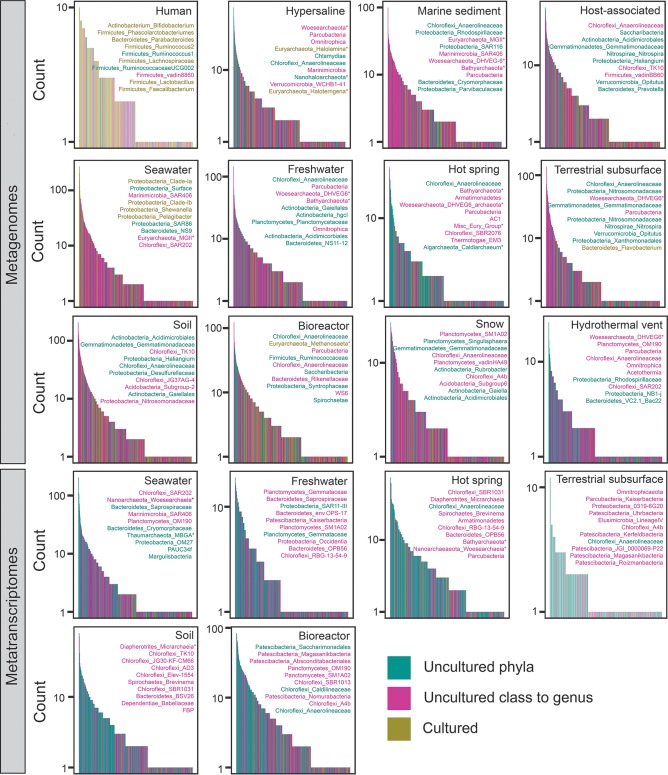
Rank abundance plots by taxonomic genus assignments for metagenomic data (top three rows) and metatranscriptomic data (bottom two rows). Listed in each box are the top 10 most abundant genera for that environment in the format of phylum_lowest identified taxonomic group, with asterisks (*) denoting archaea. Data are colored for uncultured phyla (teal), uncultured class to genus (pink) or cultured phyla (tan). Taxonomic-based genera that had sequences from multiple phylogeny-based percent identity bins were labeled with the color of the bin with the most sequences.

We tested whether only a few clades account for this global dominance of uncultured microbes. On the contrary, the metagenome data show that each category of phylogenetic novelty contained many different genera (Fig. 4). Also, genera at all levels of phylogenetic novelty were distributed throughout the rank abundance curves in all environments except for the human environment (Fig. 4). The taxonomic identities of the 10 most abundant genera differed between environments and often included genera from newly named uncultured phyla such as *Parcubacteria*, *Omnitrophica*, *Latescibacteria*, *Patescibacteria*, *Bathyarchaeota*, *Woesearchaeota*, *Armatimonadetes*, AC1, Miscellaneous Euryarchaeotal Group, *Saccharibacteria*, WS6, *Marinimicrobia*, and FBP (Fig.4). Despite having fewer overall sequences than bacteria, archaea were in the 10 most abundant genera in 8 of the 12 environments. Few of the top 10 genera in metagenomes were also in the top 10 genera in metatranscriptomes. The exception was Chloroflexi_Anaerolineaceae, which was present in the top 10 genera in both data sets for hot springs, terrestrial subsurface, and bioreactors. However, this could be an artifact of the analysis, because uncultured members of this group have not been taxonomically characterized to the genus level, so these bins may lump together many different genera that are collectively labeled “uncultured.” Some of the most abundant uncultured clades, such as “*Candidatus* Pelagibacter” in seawater, have actually been obtained in pure cultures (19), but their physiological requirements prevent them from meeting the stringent criteria required to receive an official taxonomy, such as the ability to be grown out of cell stocks. However, few other examples of such cryptically cultured organisms occurred in our data set.

Many of the top 10 genera were taxonomically identified as belonging to cultured phyla, even though we found them to be <86% similar to their nearest cultured neighbor. This is because taxonomic identification and phylogenetic identification are not identical methods. Sequences that have low similarity to culture sequences can nonetheless be given a taxonomic classification to a cultured phylum because the database used for classification also contains many instances of uncultured sequences that have previously been named part of that phylum. When genomes become available, such groups are often reassigned as phyla (1). Our results suggest that rare and abundant taxa are both cultured and uncultured, as well as bacterial and archaeal.

Our data sets likely include some amount of relic preserved DNA that can inflate diversity estimates (20). However, we do not calculate total diversity in a single sample but instead calculate occurrence frequency across many samples. Extracellular DNA from a particular taxonomic group is not likely to be abundant in the majority of samples to the exclusion of intracellular DNA from that taxonomic group. In addition, in all environments, metatranscriptomes were characterized by higher fractions of sequences from uncultured groups than the metagenomic databases were, with particularly high levels of contributions from uncultured phyla (Fig. 2). This suggests that the uncultured cells that dominate these data sets likely come from living organisms.

These results offer at least a partial explanation for “the great plate count anomaly,” which states that <1% of environmental microbial cells are culturable with standard methods (21). To update this analysis, we examined 347 experiments in 26 studies of samples from lakes, rivers, drinking water, seawater, marine and terrestrial subsurfaces, animal hosts, and soils and found a median of 0.5% culturable cells (see Table S3 in the supplemental material). The past several decades have seen considerable progress on novel culturing techniques, which have yielded higher fractions of culturable cells (25% ± 20%, *n* = 38) in fish guts (22), rice paddies (23), surface marine sediments (24, 25), agricultural soils (26), and eutrophic lakes (21). However, these studies expanded the set of cultured taxa only to novel families (24, 26), and we show that the percentages of cells from cultured families in these environments match the percentages of culturable cells reported from these studies (Table S4). Therefore, we propose that these innovative methods likely were successful at culturing viable but nonculturable cells (VBNC), which are cells from previously cultured clades that are temporarily and reversibly culture resistant (27). However, our analysis shows that a considerable fraction of cells in nonhuman environments are phylogenetically divergent, even belonging to novel phyla. We propose that representatives of these phyla resist cultivation due to more-fundamental reasons, making them phylogenetically divergent noncultured cells (PDNC). We roughly define PDNC as cells from the order level or higher with no cultured representatives. Unlike VBNC, PDNC are not dormant close relatives of cultured species that can be expected to behave like known cultures under the correct combination of growth conditions. These entire lineages may have physiologies that prevent growth in pure culture, such as dependences on syntrophic interactions (28), precise chemical or physical parameters that are difficult to maintain (24), extreme dependence on oligotrophy (19, 29, 30), or very low growth rates (31). Examples of taxa from novel phyla brought into pure culture are *Nitrosopumilus* sp., a member of the *Thaumarchaeota* phylum (29), and *Abditibacterium utsteinense*, a member of the FBP phylum (32). These required extremely low-nutrient environments and incubation times of many months to be brought into culture. Interestingly, *A. utsteinense* tolerates a wide range of antibiotics (32), so adding antibiotics to culture media may aid in the isolation of further uncultured groups. Fundamentally novel culturing techniques, possibly guided by cell physiology insights derived from genomic studies, are likely required to grow more of these highly abundant and deeply divergent clades in culture.

Given the substantial functional differences that often exist between closely related microbial species or strains, these uncultured lineages are likely to contain many novel metabolic pathways, enzyme functions, cellular structures, and physiologies (33). For instance, uncultured clades of archaea and bacteria have more genes and physiologies that are unannotatable with current databases than cultured clades (27% and 37% versus 19% and 31%, respectively; Fig. S1). In addition, rapidly growing numbers of studies are uncovering potentially important functions of uncultured clades within specific environmental contexts (14, 34–36).

10.1128/mSystems.00055-18.1 FIG S1 Percentage of genes in genomes of cultured and uncultured archaea and bacteria that are labeled “hypothetical.” Data are from IMG/JGI. Cultured, 371 archaeal species, 22,204 bacterial species; Uncultured, 107 archaeal species, 292 bacterial species. Download FIG S1, DOCX file, 0.1 MB. Copyright © 2018 Lloyd et al. 2018 Lloyd et al. This content is distributed under the terms of the Creative Commons Attribution 4.0 International license.

We conclude that uncultured taxa are abundant and alive in Earth’s microbiome, often at very high levels of phylogenetic novelty, and may harbor undiscovered functions that are important on the ecosystem level. The high proportion of sequences from uncultured groups in human-maintained bioreactors, animal and plant hosts, and soils, many of which were agricultural or municipal, shows that highly divergent novel clades not only are a feature of pristine wilderness environments but are important in engineered environments with immediate human applications as well. This suggests that results of *ex situ* experiments performed with existing microbial cultures may not represent the functions of the majority of cells *in situ*. For environmentally important VBNC, novel culture techniques are showing great success in getting them into culture (30, 37). For PDNC, novel culture-independent techniques such as genomic inference (38), label incorporation (39–41), and tracking of slow growth in a mixed population under different conditions (42) will allow the study of their physiology and ecology and guide efforts to culture them.

## MATERIALS AND METHODS

Primer-amplified sequences were obtained from Silva123Ref (www.arb-silva.de) (5), which contains chimera-checked, high-quality, >900-bp (for archaea) and >1,200-bp (for bacteria) 16S rRNA gene sequences, almost all of which represent Sanger-sequenced clone insertions from primer-amplified PCR products. The analysis yielded 952,509 bacterial and 51,608 archaeal sequences from 4,743 studies that employed a wide variety of primers. Genes that were annotated as 16S rRNA genes and were >900 bp in length were collected from the Joint Genome Institute (JGI) IMG/M database for metagenomes larger than 1 GB in total or metatranscriptomes larger than 60 Mb in total (6). These metagenomes have not undergone multiple-displacement amplification. Too few metatranscriptomes were available from humans, human-adjacent environments, rock, snow, hydrothermal vents, hypersaline environments, or marine sediments to be included. Scaffold read depths were available for metagenomes but not for metatranscriptomes.

Metagenomes and metatranscriptomes are prone to chimera production during assemblies of short reads along the highly conserved 16S rRNA gene (17). We therefore implemented uChime (43) in mothur (44) with the Silva Gold alignment to identify and remove a further 1.3% and 0.6% of possible chimeras from metagenomes and metatrancriptomes, respectively. Further chimera checks are described below. Taxonomic identifications were made for each sequence in the metagenomic and metatranscriptomic data sets in mothur (44) for alignment, preclustering, and classification to silva.nr_v132 (45). Sequences that were identified as chloroplasts, mitochondria, or eukaryotes (<1% of sequences) were removed.

BLASTn was used to determine the percent identity of each sequence to the single most closely related 16S rRNA gene sequence from cultured archaea (4,170 sequences) or bacteria (22,150 sequences) obtained from Arb-Silva. Only cultured archaea and bacteria with official names from the *International Journal of Systematic Bacteriology* or the *International Journal of Systematic and Evolutionary Microbiology* were included, excluding candidatus organisms or enrichments. Rather than relying on annotations of separate archaeal and bacterial data into metagenomes and metatrancriptomes, sequences were queried against a database with bacteria and archaea combined to get the top hit. We used a BLASTn implementation parallelized for high-performance computation (HPC-BLAST) (46) on the Beacon cluster (47) at the Joint Institute for Computational Sciences. The alignment results of HPC-BLAST are compatible with those of NCBI BLAST.

A few metagenomic and metatranscriptomic 16S rRNA gene sequences did not yield BLASTn hits and so were not considered further. For sequences with query alignment lengths of <300 bp, percent identity increased with decreasing alignment length, suggesting that these represented partial hits to small conserved regions, so they were removed from the analysis. Short query alignment lengths could also signify chimeras. Therefore, sequences with a <90% alignment length with respect to their closest cultured relative were aligned with BLASTn to the SilvaNR database, containing environmental DNA sequences. Sequences with <90% alignment to sequences in both the cultured and Silva NR databases were considered to be chimeric and were removed from analysis. This removed 6% of the metagenomic database, leaving 39,426 bacterial and 13,404 archaeal sequences from 1,504 metagenomes, as well as 7% of the metatranscriptomic database, leaving 9,396 bacterial and 3,863 archaeal sequences from 381 metatranscriptomes. Each remaining sequence was manually categorized into 1 of 14 environment types, based on user-provided metadata (Tables S1 and S2), and posted publicly at https://github.com/adsteen/quantifying_uncultured_microbes_2018.

10.1128/mSystems.00055-18.2 TABLE S1 Subtypes of environments included in each large environmental group represented in Fig. 2 and Table 1. Download Table S1, DOCX file, 0.01 MB. Copyright © 2018 Lloyd et al. 2018 Lloyd et al. This content is distributed under the terms of the Creative Commons Attribution 4.0 International license.

10.1128/mSystems.00055-18.3 TABLE S2 The number of primer-amplified sequences of bacteria and archaea from the Silva Reference Database (release 123) and metagenomes from the IMG/M metagenome database and the IMG/M metatranscriptome database. All sequences were >900 bp in length. NA, too few sequences were available to be considered. Download Table S2, DOCX file, 0.02 MB. Copyright © 2018 Lloyd et al. 2018 Lloyd et al. This content is distributed under the terms of the Creative Commons Attribution 4.0 International license.

10.1128/mSystems.00055-18.4 TABLE S3 Percent culturable cells. We identified as many studies as possible that had most-probable-number quantifications of cells plus direct cell counts. The article by Razumov (A. Razumov, Mikrobiologiia 1:131–146, 1932), referenced in the article by Staley and Konopka (21), was translated from Russian to English by Tatiana Vishnivetskaya, a microbiologist at the University of Tennessee. We have made the original text and our imperfect translation publically available at https://github.com/klloydbeaufort/great-plate-count-translated. Many of these studies used multiple types of incubations for the MPNs. In these cases, we chose the results from the experiments that produced the largest values. Although many of these studies had <1% culturability of cells, the culturing effort was often very extensive and the authors often realized the value of using oligotrophic media and waiting many months for growth to occur. For studies that had data only in plots, WebPlotDigitizer (https://automeris.io/WebPlotDigitizer/) was used to determine their values, with the exception of the study by Ludvigsen et al. (L. Ludvigsen, H.-J. Albrechtsen, D. B. Ringelberg, F. Ekelund, and T. H. Christensen, Microb Ecol 37:197–207, 1999), which had a three-dimensional (3D) plot, so the values were determined by eye. Download Table S3, DOCX file, 0.03 MB. Copyright © 2018 Lloyd et al. 2018 Lloyd et al. This content is distributed under the terms of the Creative Commons Attribution 4.0 International license.

10.1128/mSystems.00055-18.5 TABLE S4 Studies with the highest percentages of culturable cells in environmental samples determined values that were within the range of percentages of cells of cultured families in these environments. Download Table S4, DOCX file, 0.01 MB. Copyright © 2018 Lloyd et al. 2018 Lloyd et al. This content is distributed under the terms of the Creative Commons Attribution 4.0 International license.

16S rRNA gene sequences that shared more than 96.6% sequence identity with a cultured organism were considered to be in the same genus, and sequences that shared at least 86% sequence similarity were considered to be in the same phylum (12). These created “similarity bins” of cultured species to genus, uncultured genus to class, and uncultured species at the phylum level and higher. For primer-amplified, metagenomic, and metatranscriptomic data sets, the fraction of sequences in each similarity bin was calculated for a given environment. In metagenomes for which the sequence read depth was available, the fraction in each similarity bin was calculated as the sum of sequence read depths for each similarity bin within each metagenome. These values were averaged for all metagenomes in each environment.
