# Supplementary figures and images for: Phylogenetically Novel Uncultured Microbial Cells Dominate Earth Microbiomes
Source: mSystems. 2018 Sep 25;3(5):e00055-18. doi: 10.1128/mSystems.00055-18 (PMC6156271; doi:10.1128/mSystems.00055-18)

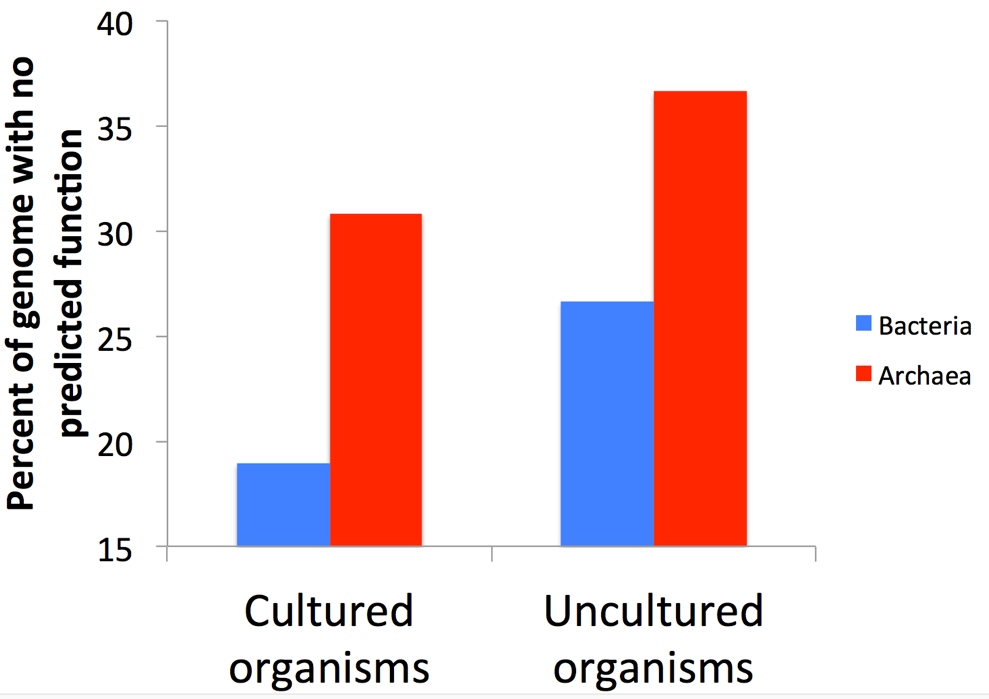

Supplement: FIG S1 [file sys004182270sf1.docx]
