# Supplementary material for: Phylogenetically Novel Uncultured Microbial Cells Dominate Earth Microbiomes
Source: mSystems. 2018 Sep 25;3(5):e00055-18. doi: 10.1128/mSystems.00055-18 (PMC6156271; doi:10.1128/mSystems.00055-18)
Supplement: TABLE S1 [file sys004182270st1.docx]

| **Environment** | **Specific types of sub-environments included** |
| --- | --- |
| Human | external and internal to the human body |
| Human-adjacent | indoor and outdoor aerosols, drinking water and biofilms, dust, manufacturing and building materials, and ballast water |
| Snow | rain, snow, glaciers, ice deposits |
| Host-associated | gastrointestinal tract, skin/hair, feces, mucus, shells, tissue and surrounding environment from livestock, laboratory model animals, seawater sponges, plants, and zoo animals |
| Bioreactor | laboratory scale reactors, compost, municipal/commercial scale waste facilities, microbial fuel cells, and digesters |
| Soil | agricultural, wetland, bogs/fens, permafrost, forests, deserts, polluted soils, and landfills |
| Rock | large datasets were only available for bacteria. Mostly endolithic communities. |
| Hydrothermal vents | water, sediment, chimneys, biofilms, deposits, plumes, in situ growth chambers, and boreholes |
| Freshwater | water, sediment, and mats from cold springs, ponds/lakes, rivers, dam reservoirs, and aquaculture |
| Terrestrial subsurface | caves, aquifers, rock fractures, mine tailings, asphalt lakes, sink holes, oil fields, and coal beds |
| Hot springs | water, sediment, precipitates, and biofilms/streamers at a range of temperature and pH |
| Seawater | all depth zones and proximities to coasts, marine estuaries/lagoons, sea ice, and polluted seawater |
| Hypersaline | crusts, lagoons, soil, and deep marine brines |
| Marine sediment | seafloor, mud volcanoes, methane seeps, salt marshes, marine estuaries, and lagoons, with a range of water and sediment depths |
