# Supplementary material for: Phylogenetically Novel Uncultured Microbial Cells Dominate Earth Microbiomes
Source: mSystems. 2018 Sep 25;3(5):e00055-18. doi: 10.1128/mSystems.00055-18 (PMC6156271; doi:10.1128/mSystems.00055-18)
Supplement: TABLE S2 [file sys004182270st2.docx]

|  | **Primer-amplified databases** | | **Metagenomic databases** | | **Metatranscriptomic databases** | |
| --- | --- | --- | --- | --- | --- | --- |
|  | **# sequences** | **# studies** | **# seq-uences** | **# studies** | **# sequences** | **# studies** |
| **Bacteria** |  |  |  |  |  |  |
| Human | 525711 | 114 | 218 | 23 | NA | NA |
| Human-adjacent | 15696 | 53 | 61 | 21 | NA | NA |
| Snow | 1085 | 33 | 1153 | 41 | NA | NA |
| Hot Springs | 4550 | 93 | 996 | 37 | 1064 | 53 |
| Host-associated | 209274 | 826 | 3420 | 147 | 69 | 25 |
| Seawater | 47158 | 281 | 3512 | 53 | 2870 | 97 |
| Freshwater | 25445 | 330 | 4684 | 118 | 766 | 41 |
| Terrestrial subsurface | 32581 | 406 | 3769 | 219 | 194 | 6 |
| Bioreactor | 50857 | 593 | 6363 | 206 | 3208 | 59 |
| Soil | 64367 | 559 | 9393 | 397 | 1226 | 52 |
| Hydrothermal vents | 5821 | 88 | 627 | 8 | NA | NA |
| Rock | 5457 | 54 | 0 | NA | NA | NA |
| Hypersaline | 3273 | 46 | 2137 | 116 | NA | NA |
| Marine sediments | 26826 | 228 | 3095 | 88 | 3 | 2 |
| **Archaea** |  |  |  |  |  |  |
| Human | NA | NA | 46 | 18 | NA | NA |
| Snow | NA | NA | 273 | 34 | NA | NA |
| Host-associated | 5298 | 121 | 998 | 138 | 21 | 15 |
| Bioreactor | 4055 | 140 | 2065 | 207 | 1118 | 52 |
| Seawater | 10911 | 78 | 1200 | 53 | 1357 | 83 |
| Freshwater | 2104 | 76 | 1700 | 121 | 259 | 51 |
| Hypersaline | 2486 | 56 | 1086 | 118 | NA | NA |
| Terrestrial subsurface | 2828 | 132 | 1178 | 207 | 141 | 5 |
| Soil | 2826 | 95 | 2877 | 390 | 511 | 52 |
| Hydrothermal vents | 2132 | 82 | 305 | 8 | NA | NA |
| Hot Springs | 2481 | 70 | 575 | 26 | 455 | 42 |
| Marine sediments | 16487 | 189 | 1097 | 88 | NA | NA |
