# Supplementary material for: Phylogenetically Novel Uncultured Microbial Cells Dominate Earth Microbiomes
Source: mSystems. 2018 Sep 25;3(5):e00055-18. doi: 10.1128/mSystems.00055-18 (PMC6156271; doi:10.1128/mSystems.00055-18)
Supplement: TABLE S3 [file sys004182270st3.docx]

| **Type of sample** | **#** | **Range** | **Reference** |
| --- | --- | --- | --- |
| Antarctic shelf sediments | 7 | 1.7-3.8 | ^1^ |
| Deep marine sediment (non-sapropel) | 2 | 0.00-0.23 | ^2^ |
| Wadden Sea sediment, below bioirrigation zone | 7 | 0.00-0.68 | ^3^ |
| Wadden Sea sediment, un-amended | 16 | 0.00-0.46 | ^4^ |
| Spruce pine soil | 3 | 0.41-2.80 | ^5^ |
| Rivers and Lakes in their natural state | 36 | 0.00-1.36 | ^6^ |
| Municipal drinking water systems | 9 | 0.04-1.11 | ^6^ |
| Terrestrial aquifer | 7 | 0.01-8.46 | ^6^ |
| Lakes in their natural state near Mt. St. Helens | 16 | 0.01-0.74 | ^7^ |
| Deep granitic groundwaters | 14 | 0.10-13.45 | ^8^ |
| Oklahoma soil | 12 | 0.00-2.08 | ^9^ |
| Forest soil | 3 | 0.00-1.00 | ^10^ |
| 36-305m away from a landfill leachate contaminated aquifer | 11 | 0.00-25.12 | ^11^ |
| Deep sea brine pool | 3 | 0.04-55.6 | ^12^ |
| Mid-ocean ridge hydrothermal vent | 3 | 0.02-0.08 | ^13^ |
| Anoxic Baltic Sea basin | 5 | 2.55-100 | ^14^ |
| Arctic sea ice, clear, and seawater | 5 | 2-62 | ^15^ |
| Seawater, neritic, oceanic, and near a pier | 15 | 0.00-13.57 | ^16^ |
| Deep marine sediments, no hydrates | 77 | 0.00-100 | ^17–20^ |
| Deep marine sediment (sapropels and surface) | 6 | 0.04-3.42 | ^2^ |
| Wadden Sea sediment, bioirrigation zone | 5 | 3.45-22.86 | ^3^ |
| Wadden Sea sediment, amended with organic matter | 8 | 0.86-10.15 | ^4^ |
| Oxic intertidal sediment | 6 | 2.50-40.03 | ^21^ |
| Rice paddy soil | 1 | 52 | ^22^ |
| Fish gut | 2 | 13.98-30 | ^23^ |
| Lakes 43-59 days after the eruption of nearby Mt. St. Helens deposited organic carbon substrate into the lakes | 23 | 1.00-81.48 | ^7^ |
| Oklahoma soil, high organic matter layer | 2 | 42.42-91.67 | ^9^ |
| Eutrophic lake | 1 | 6.8 | ^24^ |
| Landfill leachate contaminated aquifer | 2 | 31.62-50.12 | ^11^ |
| Grazed soil, heavily impacted with manure | 12 | 3.49-27.44 | ^25^ |
| Arctic sea ice, algal band | 4 | 0.89-10.40 | ^15^ |
| Oxic surface sediments of constructed wetland | 1 | 41.34 | ^26^ |
| Tide pool | 1 | 20.61 | ^16^ |
| Deep marine sediments, with hydrates | 22 | 0.11-31.12 | ^17–20^ |

**References**

1. Bowman, J. P., Mccammon, S. A., Gibson, J. A. E., Robertson, L. & Nichols, P. D. Prokaryotic Metabolic Activity and Community Structure in Antarctic Continental Shelf Sediments. **69,** 2448–2462 (2003).

2. Su, J., Engelen, B., Cypionka, H. & Sass, H. Quantitative analysis of bacterial communities from Mediterranean sapropels based on cultivation-dependent methods. **51,** 109–121 (2004).

3. Kopke, B., Wilms, R., Engelen, B., Cypionka, H. & Sass, H. Microbial diversity in coastal subsurface sediments: a cultivation approach using various electron acceptors and substrate gradients. *Appl. Environ. Microbiol.* **71,** 7819–7830 (2005).

4. Freitag, T. E., Klenke, T., Krumbein, W. E., Gerdes, G. & Prosser, J. I. Effect of anoxia and high sulphide concentrations on heterotrophic microbial communities in reduced surface sediments (Black Spots) in sandy intertidal flats of the German Wadden Sea. *FEMS Microbiol. Ecol.* **44,** 291–301 (2003).

5. Olsen, R. A. & Bakken, L. R. Viability of Soil Bacteria : Optimization of Plate-Counting Technique and Comparison Between Total Counts and Plate Counts Within Different Size Groups. 59–74 (1987).

6. Razumov, A. The direct method of calculation of bacteria in water: comparison with the Koch method. *Mikrobiologiia* **1,** 131–146 (1932).

7. Staley, J. T. & Konopka, A. Measurement of in situ activities of nonphotosynthetic microorganisms in aquatic and terrestrial habitats. *Annu. Rev. Microbiol.* **39,** 321–346 (1985).

8. Pedersen, K. & Ekendahl, S. Distribution and activity of bacteria in deep granitic groundwaters of Southeastern Sweden. *Microb. Ecol.* **20,** 37–52 (1990).

9. Bone, T. L. & Balkwill, D. L. Morphological and cultural comparison of microorganisms in surface soil and subsurface sediments at a pristine study site in Oklahoma. *Microb. Ecol.* **16,** 49–64 (1988).

10. Hirsch, P. & Rades-Rohkohl, E. Some special problems in the determination of viable counts of groundwater microorganisms. *Microb. Ecol.* **16,** 99–113 (1988).

11. Ludvigsen, L., Albrechtsen, H.-J., Ringelberg, D. B., Ekelund, F. & Christensen, T. H. Distribution and composition of microbial populations in a landfill leachate contaminated aquifer (Grindsted, Denmark). *Microb. Ecol.* **37,** 197–207 (1999).

12. Sass, A. M., Sass, H., Coolen, M. J. L. & Cypionka, H. Microbial communities in the chemocline of a hypersaline deep-sea Basin (Urania Basin, Mediterranean Sea). *Appl. Environ. Microbiol.* **67,** 5392–5402 (2001).

13. Huber, J. A., Butterfield, D. A. & Baross, J. A. Temporal changes in archaeal diversity and chemistry in a mid-ocean ridge subseafloor habitat. *Appl. Environ. Microbiol.* **68,** 1585–1594 (2002).

14. Bruns, A., Cypionka, H. & Overmann, J. Cyclic AMP and acyl homoserine lactones increase the cultivation efficiency of heterotrophic bacteria from the Central Baltic Sea. *Appl. Environ. Microbiol.* **68,** 3978–3987 (2002).

15. Junge, K., Imhoff, F., Staley, T. & Deming, J. W. Phylogenetic diversity of numerically important Arctic sea-ice bacteria cultured at subzero temperature. *Microb. Ecol.* **43,** 315–328 (2002).

16. Jannasch, H. W. & Jones, G. E. Bacterial populations in sea water as determined by different methods of enumeration. *Limnol. Oceanogr.* **4,** 128–139 (1959).

17. Cragg, B., Harvey, S., Fry, J., Herbert, R. & Parkes, R. Bacterial Biomass and Activity in the Deep Sediment Layers of the Japan Sea, Hole 798B. *Proc. Ocean Drill. Progr.* **127/128,** 761–776 (1992).

18. Cragg, B. A. *et al.* Bacterial populations and processes in sediments containing gas hydrates (ODP Leg 146: Cascadia Margin). *Earth Planet. Sci. Lett.* **139,** 497–507 (1996).

19. Wellsbury, P., Goodman, K., Cragg, B. a & Parkes, R. J. The geomicrobiology of deep marine sediments from Blake Ridge containing methane hydrate (Sites 994, 995, and 997). *Proc. Ocean Drill. Progr. Sci. Results* **164,** 379–391 (2000).

20. Wellsbury, P., Mather, I. & Parkes, R. J. Geomicrobiology of deep, low organic carbon sediments in the Woodlark Basin, Pacific Ocean. *FEMS Microbiol. Ecol.* **42,** 59–70 (2002).

21. Kaeberlein, T., Lewis, K. & Epstein, S. S. Isolating ‘uncultivable’ microorganisms in pure culture in a simulated natural environment. *Science* **296,** 1127–9 (2002).

22. Chin, K., Hahn, D., Hengstmann, U. L. F., Liesack, W. & Janssen, P. H. Characterization and identiﬁcation of numerically abundant culturable bacteria from the anoxic bulk soil of rice paddy microcosms. *Appl. Environ. Microbiol.* **65,** 5042–5049 (1999).

23. Yano, Y., Nakayama, A. & Yoshida, K. Distribution of Polyunsaturated Fatty Acids in Bacteria Present in Intestines of Deep-Sea Fish and Shallow-Sea Poikilothermic Animals. **63,** 2572–2577 (1997).

24. Bartscht, K., Cypionka, H. & Overmann, J. Evaluation of cell activity and of methods for the cultivation of bacteria from a natural lake community. *FEMS Microbiol. Ecol.* **28,** 249–259 (1999).

25. Janssen, P. H., Yates, P. S., Grinton, B. E., Taylor, P. M. & Sait, M. Improved culturability of soil bacteria and isolation in pure culture of novel members of the divisions Acidobacteria, Actinobacteria, Proteobacteria, and Verrucomicrobia. *Appl. Environ. Microbiol.* **68,** 2391–2396 (2002).

26. Nicomrat, D., Dick, W. a & Tuovinen, O. H. Microbial populations identified by fluorescence in situ hybridization in a constructed wetland treating acid coal mine drainage. *J. Environ. Qual.* **35,** 1329–1337 (2006).
