# Supplementary material for: Phylogenetically Novel Uncultured Microbial Cells Dominate Earth Microbiomes
Source: mSystems. 2018 Sep 25;3(5):e00055-18. doi: 10.1128/mSystems.00055-18 (PMC6156271; doi:10.1128/mSystems.00055-18)
Supplement: TABLE S4 [file sys004182270st4.docx]

|  | **Published culturing studies** | | | **Our metagenome analysis** | |
| --- | --- | --- | --- | --- | --- |
| **Environment** | **Refs for culture studies** | **% culturable cells ± std dev** | **Number of culture experi-ments** | **% seqs from cultured familes ± std dev^a^** | **Number of meta-genomic samples** |
| **Surface marine sediment** | ^3,21^ | 13 ± 9 | 10 | 26 ± 12 | 81 |
| **Host-associated** | ^22,23^ | 31 ± 19 | 3 | 31 ± 11 | 87 |
| **Eutrophic lakes** | ^7^ | 37 ± 25 | 13 | 31 ± 10 | 5 |
| **Soil** | ^25^ | 14 ± 9 | 12 | 30 ± 12 | 297 |

**^a^Average of percentages of metagenomic 16S rRNA gene sequences from cultured families (>92.5% to closest cultured relative) in each metagenomic sample.**
